# Malignant Müllerian Mixed Tumor of the Uterine Cervix with a Small Cell Neuroendocrine Carcinoma Component

**DOI:** 10.1155/2013/630859

**Published:** 2013-02-26

**Authors:** Satoru Munakata, Emi Iwai, Tomohito Tanaka, Michihiko Nakamura, Takayoshi Kanda

**Affiliations:** ^1^Department of Pathology, Osaka Minami Medical Center, 2-1 Kidohigashi, Kawachinagano, Osaka 586-8521, Japan; ^2^Department of Pathology, Sakai City Hospital, 2-7, Daigakucho, Takatsuki, Osaka 569-8686, Japan; ^3^Department of Obstetrics and Gynecology, Osaka Minami Medical Center, 2-1 Kidohigashi, Kawachinagano, Osaka 586-8521, Japan; ^4^Department of Obstetrics and Gynecology, Osaka Medical College, 2-7, Daigakucho, Takatsuki, Osaka 569-8686, Japan

## Abstract

Malignant Müllerian mixed tumors (MMMTs) of the uterine cervix are extremely rare, accounting for 0.005% of all cervical malignancies. To date, only approximately 50 well-documented cases have been reported. Although several epithelial components have been described in cervical MMMTs, small cell neuroendocrine carcinoma (SCC) has not appeared in the English literature. We present a 43-year-old woman, para 2 gravida 2, who had MMMT with SCC and rhabdomyosarcoma components in the uterine cervix. She was referred to our hospital because of a cervical mass with an abnormal Pap smear result. Cervical biopsy revealed SCC. After neoadjuvant chemotherapy with balloon-occluded arterial infusion, she underwent type II radical hysterectomy with pelvic lymphadenectomy. Histological analysis revealed that the cervical tumor comprised SCC and rhabdomyosarcoma components. Genotype analysis indicated human papillomavirus type 18. She underwent concurrent chemoradiation therapy. The patient had been free of the disease and showed no evidence of recurrence 38 months after operation.

## 1. Introduction

 Malignant Müllerian mixed tumors (MMMTs, carcinosarcomas) of the uterine cervix are extremely rare, accounting for 0.005% of all cervical malignancies [1] and are known to behave aggressively. To date, only approximately 50 well-documented cases have been reported [1–6]. Because of their rarity, no consensus has been reached regarding treatment, prognosis, and outcome [2, 6].

 Histologically, MMMTs comprise epithelial and stromal components, with many combinations. However, to our knowledge, no case of combined small cell neuroendocrine carcinoma (SCC) and rhabdomyosarcoma has been reported yet.

 We report a case of an MMMT with rare histological features that was successfully treated with neoadjuvant chemotherapy using balloon-occluded arterial infusion (BOAI), surgery, and concurrent chemoradiation therapy.

## 2. Case Report

 A 43-year-old woman, para 2 gravida 2, was referred to the Department of Obstetrics and Gynecology at the Osaka Minami Medical Center because of a uterine cervical mass with an abnormal Pap smear result. On magnetic resonance imaging, the cervical mass measured 67 × 54 mm at presentation. Based on the result of the histological examination, using a punch biopsy specimen, SCC was diagnosed, and neoadjuvant chemotherapy with BOAI was performed twice with cisplatin (100 mg), mitomycin C (10 mg), and pirarubicin (50 mg). The tumor was downsized to 24 × 16 mm in 2 months. She subsequently underwent type II radical hysterectomy with pelvic lymphadenectomy. For adjuvant therapy, she underwent 5 courses of chemotherapy with 50 mg cisplatin and 40 Gy whole pelvic irradiation. Her follow-up examination results obtained 38 months after the operation were unremarkable.

## 3. Pathologic Findings

**Figure 1:**
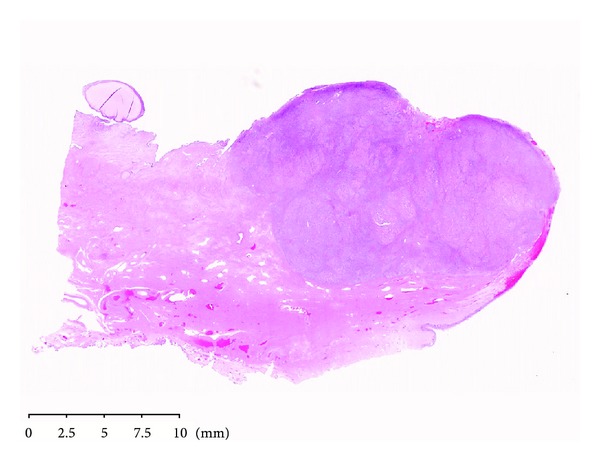
Neoplastic proliferation was located in the stroma of the uterine cervix (H&E original magnification ×0.4, NanoZoomer Digital Pathology image).

**Figure 2:**
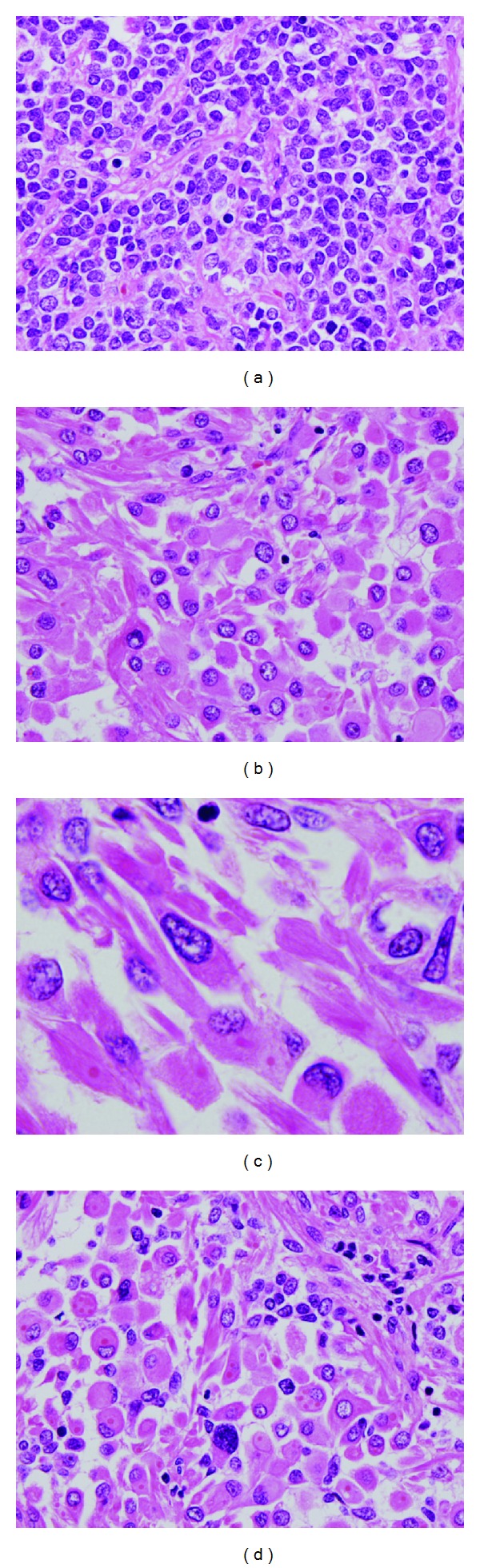
Microscopic findings. (a) Component A showed small round cell proliferation. (b) Component B consisted of large, round-to-polyhedral cells with abundant eosinophilic cytoplasm. (c) Cross-striation was observed in these cells. (d) Cells of two components intermingled intimately (H&E original magnification ×40 for (a), (b), and (d) and ×100 for (c)).

 Grossly, the anterior lip of the uterine cervix was occupied by the 27 × 23 mm hard round mass. On cross-sectional examination, the tumor was whitish yellow and solid. Microscopically, the tumor was located in the stroma of the uterine cervix (Figure 1). The tumor composed 2 sets of components. Component A included sheets, nests, and trabeculae of small round-to-ovoid cells with scanty cytoplasm. The nuclei exhibited a coarse chromatin pattern (Figure 2(a)). There was occasional nuclear molding. Component B included large, round-to-polyhedral cells with abundant eosinophilic cytoplasm (Figure 2(b)). Some of these cells had spindle, strap-like, and fibrillary cytoplasm. Cross-striation was occasionally observed (Figure 2(c)). The cells contained enlarged irregular nuclei with prominent nucleoli. Sporadic multinucleated cells were observed. Occasionally, these 2 components intermingled intimately (Figure 2(d)). Necrotic foci and, in one of the right external iliac lymph nodes, lymph node metastasis were observed.

**Figure 3:**
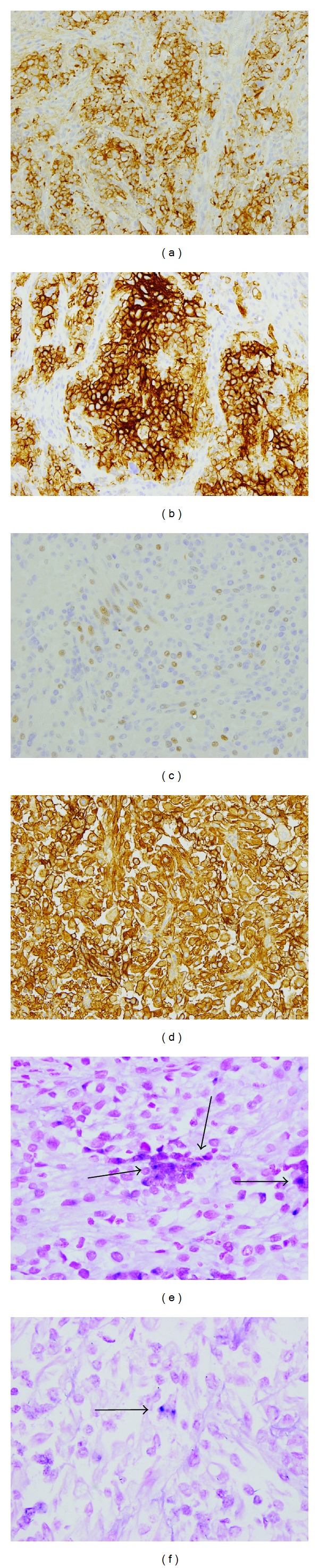
Immunohistochemical findings. Cells in component A showed positive staining for (a) synaptophysin and (b) CD56. Cells in component B were positive for (c) myogenin and (d) CD56. In situ hybridization for HPV type 16/18 showed dot-like staining consistent with viral integration pattern in the nuclei (arrow) of (e) component A and (f) component B (original magnification ×20 for (a)–(d) and ×40 for (e) and (f)).

The pathological stage of the tumor was ypT1b1 N1 M0. It was classified as stage Ib1 according to the criteria of the International Federation of Gynecologists and Obstetricians (2008) [7]. Immunohistochemical analysis was performed for cytokeratin (CK), CK7, CK20, epithelial membrane antigen (EMA), vimentin, desmin, *α*-smooth muscle actin, S-100, MyoD1, myogenin, neuron-specific enolase (NSE), synaptophysin, chromogranin A, CD56, CD10, CD99, carcinoembryonic antigen, p53, p63, p16, and Ki-67. In component A, the tumor cells were positive for EMA, NSE, synaptophysin (Figure 3(a)), chromogranin A, CD56 (Figure 3(b)), p53, p63, and p16 and negative for the rest of the antibodies. The Ki-67 labeling index was 9%. In component B, the tumor cells were positive for desmin, MyoD1, myogenin (Figure 3(c)), CD56 (Figure 3(d)), and CD10 and negative for the other antibodies. The Ki-67 labeling index was 8%. Based on these results, component A was diagnosed as SCC and component B as rhabdomyosarcoma. Component A should be differentiated from nonkeratinizing squamous cell carcinoma with small cells. Tumor cells in component A showed nuclear molding, and no squamous differentiation was found. Also, this component exhibited obvious neuroendocrine differentiation, proved by several neuroendocrine markers. Therefore, this component was diagnosed as SCC. The in situ hybridization specimen revealed dot-like staining with a viral integration pattern in the nuclei of both components (Figures 3(e) and 3(f)). Human papillomavirus (HPV) genotyping was performed with GeneSquare HPV analysis (Kurabo, Neyagawa, Japan) using a paraffin-embedded tissue sample. The test result demonstrated the presence of HPV type 18.

## 4. Discussion

 MMMTs of the uterine cervix are extremely rare. To date, only approximately 50 well-documented cases have been reported [1–6], in which the patients' ages ranged from 12 to 94 years (median, 64.5 years; mean, 60.2 years). The most common symptom is abnormal vaginal bleeding. A cervical mass and an abnormal Pap smear test result are also common initial manifestations [2, 4]. Consistent with previous case reports, our patient was referred to our hospital because of a cervical mass and an abnormal Pap smear result. Grossly, cervical tumors range from 1.1 cm to 10.0 cm in maximal dimension [2]. They form polypoid, fungating, fungiform, papillary, or pedunculated soft-to-firm masses. Microscopically, the documented epithelial components of cervical MMMT are squamous cell carcinoma (SqCC), adenoid basal carcinoma, adenoid cystic carcinoma, adenocarcinoma, serous adenocarcinoma, and poorly differentiated carcinoma. In contrast to the uterine counterpart, whose epithelial component is usually adenocarcinoma, a cervical MMMT often has SqCC components. To our knowledge, however, MMMTs with a SCC component have not yet been reported; this is the first paper describing SCC as an epithelial component of cervical MMMT. Our case is that of a heterologous MMMT due to a rhabdomyosarcoma presenting as its stromal component. As in our case, neuroendocrine carcinomas are rarely combined with sarcomas with skeletal muscle differentiation. To date, only 10 such cases have been reported [8–10]. These tumors originate from various anatomical sites, including the skin, nasal cavity, larynx, lung, small intestine, anorectal region, pancreas, and urinary bladder. However, no studies have reported such tumors originating from the uterine cervix, and ours is the first to report such case. By definition, these tumors should be classified as a carcinosarcoma because of their carcinoma and sarcoma components. However, the histogenesis of MMMTs is controversial. A previous study [11] using molecular analysis suggested that MMMTs have a monoclonal nature. Recently, Grayson et al. [3] found an HPV gene from cervical MMMTs, including 3 neoplasms in which HPV type 16 was detected. In contrast, HPV type 18 was found in our case. This finding is interesting in that it is consistent with those of previous studies [12, 13] which specified that HPV type 18 was predominantly detected in cervical SCCs. Hence, the histogenesis of our tumor is likely to be closer to that of SCCs rather than to that of MMMTs. Epithelial and mesenchymal components were admixed intimately in our case, with both components showing immunohistochemical overlap of CK, CK7, CK20, vimentin, and CD56. Based on these findings, the rhabdomyosarcomatous component in our case is likely to be metaplastic; hence, the metaplastic carcinoma theory could be applied to our case of MMMT [3, 14]. However, a clonality analysis should be performed to confirm our hypothesis.

 The prognosis in cervical MMMTs depends on the clinical stage of the disease and is better than that in the uterine counterpart because of their early detection in many cases [2, 6]. However, it is too early to be conclusive at present because of the limited number of patients and short follow-up period in previous studies. The follow-up duration in most of the cases reported was shorter than 5 years [6]. Iida et al. [5] reported the case of a patient with stage Ib1 tumor who died immediately, after 17 months of operation and radiotherapy. Cervical MMMTs are managed with surgery, chemotherapy, and radiotherapy. Cisplatin-based chemotherapy was reported to yield a high response rate [6]. The patient prognosis in cervical SCCs is even poorer than that in MMMTs. The overall 5-year disease survival rates of patients with stage I-IIA and stage IIB-IV SCCs were 36.8% and 8.9%, respectively [15]. The therapeutic modalities for SCC of the uterine cervix are surgery, chemotherapy, and radiation. In the study by Cohen et al. [15], early-stage (I-IIA) disease, chemotherapy, and radical hysterectomy were independent prognostic factors for improved survival. When viewed from the standpoint of SCC, our patient had favorable prognostic factors.

 MMMT and SCC have an aggressive nature. Fortunately, our case with pelvic lymph node metastasis was diagnosed at its early clinical stage and was well managed with neoadjuvant chemotherapy with BOAI, which several authors [16, 17] reported as effective for advanced cervical cancer when combined with surgery and concurrent chemoradiation therapy. 

 In summary, we present a case of cervical MMMT with SCC and rhabdomyosarcoma components. To our knowledge, this is the first paper of cervical MMMT with an extremely rare association. Despite its aggressive nature, our case was successfully managed with the combination of neoadjuvant chemotherapy using BOAI, surgery, and concurrent chemoradiation therapy. However, further follow-up study is necessary.
